# Baseline Tumor-Specific Prognosis in Early-Stage Hepatocellular Carcinoma: Time-Dependent Role of Biomarker Profile and Modified ALBI Grade

**DOI:** 10.3390/cancers18132073

**Published:** 2026-06-26

**Authors:** Kelley Núñez, Juan Gimenez, Ari J. Cohen, Jeffrey Burton, Tyler Sandow, Paul Thevenot

**Affiliations:** 1 Institute of Translational Research, Ochsner Health System, New Orleans, LA 70121, USA; kelley.nunez@ochsner.org; 2 Interventional Radiology, Ochsner Health System, New Orleans, LA 70121, USA; juan.gimenez@ochsner.org (J.G.); tyler.sandow@ochsner.org (T.S.); 3 Multi-Organ Transplant Institute, Ochsner Health System, New Orleans, LA 70121, USA; acohen@ochsner.org; 4 Faculty of Medicine, University of Queensland, Brisbane, QLD 4006, Australia; 5 Ochsner Center for Outcomes Research, Ochsner Health System, New Orleans, LA 70121, USA

**Keywords:** hepatocellular carcinoma, AFP, AFP-L3, DCP, mALBI

## Abstract

**Background/Objectives**: Identifying aggressive tumor biology within early-stage hepatocellular carcinoma (HCC) remains challenging. Scores based on liver function, systemic inflammation, and HCC biomarkers have been linked to overall survival prognosis; however, the combined ability of these scores to assess tumor-specific prognosis in early-stage disease is unclear. In this single-center, prospective study, biomarker profiling with AFP, AFP-L3, and DCP, along with modified albumin–bilirubin (mALBI), and neutrophil–lymphocyte ratio (NLR)/platelet–lymphocyte ratios (PLRs) were evaluated to determine their prognostic role in assessing clinical manifestations of aggressive biology by stratifying HCC progression risk. **Methods**: Indices and biomarkers were assessed at BCLC-A-stage HCC diagnosis and prior to liver-directed therapy (LDT). The primary prospective study endpoint was time-to-advanced-stage tumor progression (TTP). **Results**: The cohort included 232 patients diagnosed with early-stage HCC who underwent treatment with LDT. A multivariate model revealed that mALBI grade (*p* = 0.021), cumulative lesion size (*p* = 0.005), and elevations in HCC biomarkers (*p* < 0.001) were associated with TTP. Biomarker profile stratified TTP (*p* < 0.001) in which patients with complex profiles (3+) had 1-year progression risks of 69%. The biomarker system retained the ability to stratify TTP within small (≤3 cm) and large (>3 cm) cumulative tumor burden (*p* < 0.001, *p* = 0.005). While PLR was not prognostic for TTP, NLR disappeared from the multivariate model and mALBI stratified long-term progression risk (*p* = 0.003). In low-complex biomarker patients (0–1+), mALBI stratified progression risk (*p* = 0.001). **Conclusions**: Multi-positive biomarker profiling in early-stage HCC identifies a population with clinical manifestations of aggressive tumor biology at high risk of rapid post-treatment disease progression that may benefit from more aggressive treatment approaches. In patients with low-risk biomarker profiles (0–1+), mALBI can assess longer-term (>1-year) post-treatment disease progression risk, while scores based on systemic inflammation were not associated with tumor-restricted outcomes.

## Simple Summary

Blood-based methods to identify aggressive tumor biology in early-stage hepatocellular carcinoma (HCC) are greatly needed. Numerous indices that assess liver function (modified albumin–bilirubin [mALBI]), systemic inflammation (neutrophile–lymphocyte [NLR] and platelet–lymphocyte ratios [PLR]), as well as HCC biomarkers (alpha fetoprotein [AFP], AFP-Lens culinaris agglutinin [AFP-L3] and des-gamma-carboxy prothrombin [DCP]) are effective at predicting overall survival. In this study, HCC biomarkers, AFP, AFP-L3, and DCP, stratified progression risk with complex biomarker profiles (3+) with the highest 1-year progression risks (69%) in early-stage HCC following treatment with liver-directed therapy. In low-complex biomarker profiles (0–1+), mALBI could assess long-term (>1-year) progression risk. However, neither NLR nor PLR was able to stratify progression risk in a multivariate model. These findings suggest that multi-positive biomarker profiling can identify patients at risk of rapid progression following treatment.

## 1. Introduction

HCC remains one of the leading causes of cancer mortality worldwide [1]. Most HCC develops in a background of existing liver disease, which, in the case of cirrhosis, may exacerbate performance status decline and quality of life. HCC treatment can also exacerbate liver dysfunction and negatively influence treatment outcomes [2,3] and overall prognosis [4]. Liver-directed therapies (LDTs) are used both as curative treatments and as a bridge to delay tumor progression until surgical intervention. While LDT is an effective treatment option for early-stage HCC, 25% of these patients experience tumor progression [5,6] due to unanticipated aggressive tumor biology following LDT and may benefit from alternative treatment strategies.

Several composite scores for assessing liver function or systemic inflammation have been effective in stratifying overall survival (OS) prognosis in HCC, particularly in advanced-stage disease. The albumin–bilirubin (ALBI) grade serves as an indicator of hepatic functional reserve [7] and has been utilized to stratify OS while also serving as a prognostic indicator for response to LDT (see review [8]). The modified ALBI (mALBI) [7] further refines ALBI grade 2 while providing an additional level of OS stratification [9], although its ability to assess LDT prognosis remains unclear. The neutrophil-to-lymphocyte ratio (NLR) and platelet-to-lymphocyte ratio (PLR) are used to measure systemic inflammation, primarily driven by neutropenia and thrombocytosis, and are routinely used to stratify OS prognosis [10,11,12]. However, studies that evaluate the effectiveness of the NLR and PLR in response to LDT in early-stage disease are scarce, and evidence supporting their link to aggressive biology or disease progression risk is limited.

While the ALBI and classifications based on tumor burden can effectively stage HCC, stage-dependent assessments of aggressive biology have largely focused on alpha-fetoprotein (AFP) expression levels. More recently, AFP in combination with AFP-Lens culinaris agglutinin (AFP-L3) and des-gamma-carboxy prothrombin (DCP) has shown an improved capacity for HCC diagnosis [13] as well as an ability to stratify aggressive tumor biology [14,15]. The AFP, AFP-L3, and DCP panel has received FDA breakthrough device designation for HCC surveillance, but its clinical utilization beyond diagnosis has been limited. Several prospective studies have used the panel to establish an HCC biomarker phenotype and treatment prognosis in early- and intermediate-stage disease [16,17,18]. Importantly, these studies in early-stage disease can reduce the confounding influence of end-stage liver disease and illuminate the direct relationships between the HCC biomarkers, hepatic/inflammatory scores, and tumor-specific treatment outcomes.

This study utilizes a single-center, prospective, baseline HCC biomarker phenotyping study to evaluate the ability of tumor-specific (AFP, AFP-L3, DCP), liver-specific (mALBI), and systemic inflammatory indices (NLR/PLR) to assess tumor progression for advanced-stage risk in early-stage disease undergoing LDT.

## 2. Materials and Methods

### 2.1. Study Population

Written informed consent, with approval by the Institutional Review Board (# 2016.131.B), was obtained in accordance with the ethical guidelines set forth by the 1975 Declaration of Helsinki. All patients were reviewed through a single institutional multi-disciplinary tumor board with enrollment dates that spanned from August 2016 to May 2025. Inclusion criteria were: (i) HCC diagnosis confirmed by biopsy or triple-phase imaging according to the Liver Imaging-Reporting and Data System (v2018); (ii) unresectable HCC determined by surgical oncologists for inadequate liver function due to extensive cirrhosis, poor synthetic function, and prior hepatic decompensation; (iii) ≥18 years of age; (iv) BCLC-A-stage disease; and (v) scheduled to receive first-cycle LDT. Exclusion criteria included: (i) missing baseline sample, (ii) warfarin use, and (iii) co-malignancies.

### 2.2. Clinical Data, Liver Function, and Systemic Inflammation Index Calculations

Clinical variables were exported from the electronic medical record and included demographics, complete metabolic panel, complete blood count panel, and model of end-stage liver disease scores. All laboratory values were obtained from pre-treatment lab orders prior to the first-cycle LDT.

ALBI and modified ALBI grades were calculated as previously described [19,20]. NLR was calculated by dividing the absolute neutrophil count (10^3^/μL) by the absolute lymphocyte count (10^3^/μL). PLR was calculated by dividing the platelet count (10^3^/μL) by the absolute lymphocyte count (10^3^/μL). All indices were calculated from laboratory values taken prior to first-cycle LDT.

### 2.3. Liver-Directed Therapies

Three different liver-directed therapy (LDT) modalities were available for treatment and included: doxorubicin eluting bead transarterial chemoembolization (DEB-TACE), microwave ablation (MWA), and Yttrium-90 (^90^ Y). All LDTs were performed within a single healthcare system (Ochsner Health). Institutional criteria received LDT were: (i) BCLC-A, (ii) ECOG 0–1, (iii) Child–Pugh score A-B or sufficient preserved liver function determined by the tumor board. LDT modality selection was determined by the interventional radiology program treatment algorithm, incorporating both tumor size and location, with the preference of the interventional radiology provider. All LDTs were performed with curative intent. Radioembolization with ^90^Y was performed with curative intent with doses exceeding established ablative thresholds. All DEB-TACE procedures were also performed with the goal of super-selective treatment of all vessels, targeting a complete radiographic response to serve as a potential curative bridge to liver transplantation.

### 2.4. Biomarker Assessment

Peripheral blood was collected into sodium citrate cell preparation tubes (BD Biosciences, East Rutherford, NJ, USA) immediately prior to first-cycle LDT. Blood specimens were processed according to the manufacturer’s protocol to obtain plasma that was immediately stored at −80 °C until biomarker measurements. Levels of the biomarkers AFP, AFP-L3, and DCP were measured using μTASWakoi30 (FUJIFILM Wako Diagnostics, Mountain View, CA, USA). Minimum detectable ranges for each biomarker were as follows: AFP > 4.5 ng/mL, AFP-L3 > 0.5%, and DCP > 1.5 ng/mL. Biomarker values at the minimum level of detection were recorded as the minimum value. Each biomarker was deemed positive if the expression level exceeded previously established thresholds: AFP > 20 ng/mL, AFP-L3 > 15%, and DCP > 7.5 ng/mL [21].

### 2.5. Study Outcomes

The primary study endpoint was time-to-progression (TTP), defined as the time from first-cycle LDT to stage of progression from BCLC-A to BCLC-C based on tumor progression and the development of extrahepatic metastasis, macrovascular invasion, or infiltrative or diffuse bilobular HCC. TTP was censored for the following: >6 months without follow-up or surveillance imaging, all-cause mortality, liver transplantation, or free-of-stage progression to BCLC-C at the time of data analysis (1 August 2026). Censoring date was defined as the date of the most recent imaging appointment.

### 2.6. Statistical Analysis

Data analysis was performed using JMP version 18 (JMP Statistical Discovery, Cary, NC, USA). All graphical output was generated using GraphPad Prism version 10.6.0 (GraphPad Software Inc., San Diego, CA, USA). Continuous variables were displayed as medians with interquartile ranges (IQRs) and categorical variables as a percentage of the total cohort. Univariate Cox proportional hazards models were used to calculate hazard ratios for TTP. Variables from the univariate analysis with *p* < 0.050 were included in the multivariate Cox proportional hazards models for TTP. Kaplan–Meier survival curves were generated in GraphPad Prism and compared using log-rank tests. Curves were trimmed to the nearest 6-month timepoint after the at-risk population fell below 10% of the total population being analyzed.

## 3. Results

### 3.1. Cohort Characteristics

A total of 232 patients with a recent unresectable HCC diagnosis of BCLC-A disease were included in the study. Demographics at diagnosis are shown in Table 1. The median age was 64 years, with 70% (162/232) male and 78% (180/232) Caucasian. The most common etiology of liver disease was viral hepatitis (63%, 145/232). Most patients had an ECOG score of 0 (71%, 161/232) and were Child–Pugh A (61%, 141/232). Preserved liver function assessed through ALBI and mALBI scores revealed that most patients were ALBI grade 2 (66%, 153/232) or mALBI grade 2b, making up 44% of the cohort (101/232). The median MELD 3.0 of the cohort was 10.

Within BCLC-A sub-staging, 85% of patients had solitary disease (197/232). The median primary lesion size was 2.8 cm, with a median cumulative lesion size of 3.3 cm. Median levels of the HCC biomarkers were as follows: AFP of 7.7 ng/mL, AFP-L3 of 5.3%, and DCP of 2.6 ng/mL. After thresholding positive expression levels for each biomarker, most patients were negative for all three biomarkers at the time of diagnosis (46%, 107/232), while 27% (63/232) were positive for one biomarker (1+), 18% (42/232) were double positive (2+), and 9% (20/232) were positive for all three biomarkers (3+). All patients received LDT as the first cycle of treatment, with 59% (137/232) treated with ^90^Y, 19% (44/232) treated with DEB-TACE, and 22% (51/232) treated with MWA.

### 3.2. Factors and Indices Associated with Time to Progression from Early to Advanced Stages

Univariate Cox analysis was performed to identify variables and composite indices associated with TTP risk while retaining BCLC-A intrinsic measures of tumor burden (size and burden) associated with HCC prognosis. Endpoint data is shown in Supplemental Table S1. The multi-level variables mALBI, cumulative size, and biomarker expression profiles were individually analyzed by univariate Cox to identify the optimal subgrouping strategy to isolate statistical differences (Supplemental Table S2). The comprehensive results for all baseline variables are summarized in Table 2. As previously highlighted, NLR and PLR lack consensus thresholds in the literature and are often derived from cohort-specific median/mean splits or internally optimized thresholds following logistic regression to an outcome of interest. A panel of representative NLR and PLR thresholds from these HCC studies, along with the cohort-specific internal median split, was evaluated by Cox univariate against TTP (Supplemental Tables S3 and S4). None of the PLR thresholds investigated were able to stratify TTP risk, while two NLR cutoffs did. From the results in Table 2, only albumin (continuous), mALBI grade (1–2a vs. 2b–3), NLR (2.81 and 3.0), tumor size (index lesion size and cumulative lesion size), and stepwise increases in biomarker expression profile were associated with TTP. In the multivariate model, mALBI grade (1–2a vs. 2b–3), cumulative lesion size (≤ and > 3 cm), and elevations in HCC biomarkers (0–1+ vs. 2+ vs. 3+) remained associated with TTP (Table 2). Albumin was not included, as this was incorporated into the mALBI grade.

**Figure 1:**
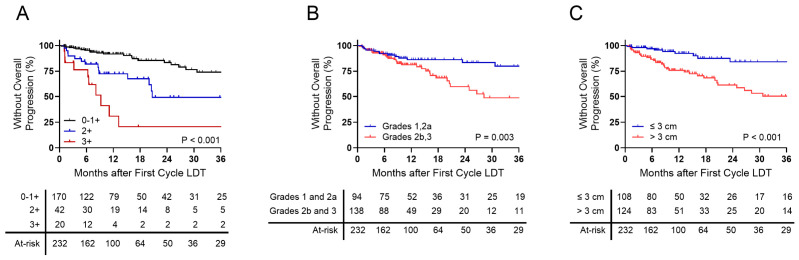
Overall time-to-progression based on HCC biomarker expression profile and mALBI grade. Overall TTP following the first cycle of liver-directed therapy based on (**A**) HCC biomarker profiles, (**B**) mALBI grade, and (**C**) cumulative lesion size grouped at the time of HCC diagnosis.

The TTP prognostic factors that persisted through multivariate analysis are visualized as time-to-event curves in Figure 1, with failure plots shown in Supplemental Figure S1. A triple positive (3+) AFP, AFP-L3, and DCP profile was associated with the greatest risk of HCC progression, with a dismal median TTP of 9.2 months (3.2–13.2) compared to 20.6 months in patients with elevations in two biomarkers (2+) and the 0–1+ biomarker group, which did not reach a median TTP (Figure 1A). Stratification was pronounced at 1 year after treatment initiation, where progression rates were 69% in 3+ profiles compared to 28% for 2+ profiles, and 8% for 0–1+ biomarkers. In contrast, mALBI resulted in delayed stratification beginning 1 year after treatment initiation (Figure 1B). The median TTP for patients with worse mALBI grades (grades 2b–3) was 28 months (20–65 months) compared to the median not reached in patients with grades 1-2a (Figure 1B). Post-treatment progression rates for mALBI grades 2b–3 were nearly doubled compared to grades 1–2a at 2 and 3 years following initial LDT (grades 2b–3: 41% and 51%; grades 1–2a: 17% and 20%). Cumulative tumor burden exceeding 3 cm was also associated with higher progression risk, with a median TTP of 65 months (21—not reached) compared to small burdens (≤3 cm), where the median TTP was not reached. Progression rates at 1 and 2 years when tumor burden surpassed 3 cm was 24% and 39%, compared to 8% and 16% if the burden was below 3 cm.

### 3.3. HCC Biomarker Expression Profile and Progression Risk in BCLC-A Based on Cumulative Lesion Size

Given that the cumulative burden was maintained in the multivariate model, the potential for colinear influences between tumor burden and biomarker profile complexity or mALBI progression was evaluated (Supplemental Table S5). While mALBI profiles were consistent across small (≤3 cm) and larger (>3 cm) cumulative burdens, complex biomarker profiles, specifically the triple positive (3+) profile, were more frequent with a larger cumulative burden. As observed between mALBI and cumulative burden, there was no relationship between mALBI grade severity and biomarker profile.

**Figure 2:**
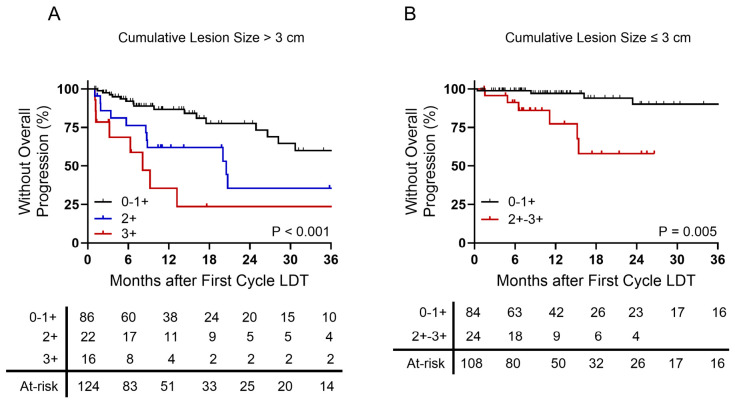
Overall time-to-progression by cumulative lesion size based on the number of positive HCC biomarkers. (**A**) Overall TTP in patients with initial cumulative lesion sizes of either (**A**) >3 cm or (**B**) ≤3 cm based on HCC biomarker profiles at the time of HCC diagnosis.

However, the increased frequency of complex (3+) biomarker profiles with higher BCLC-A cumulative burden at diagnosis did not compromise TTP outcome stratifications (Figure 2, failure plots shown in Supplemental Figure S2). The TTP profile based on the biomarker profile in patients with larger cumulative burden (Figure 2A) mirrored the overall cohort (Figure 1A), with median progression rates of 20 months for 2+ biomarkers and 8 months with 3+ biomarkers. This corresponded to 1-year progression rates of 38% for 2+ biomarkers and 65% for 3+ biomarkers compared to 13% in patients with 0–1+ biomarkers. In small cumulative BCLC-A burden, the TTP profiles for 2+ and 3+ biomarkers were similar but collectively yielded a significant difference compared to 0–1+ biomarker profiles (Figure 2B). Despite limited early-stage tumor burden, patients with 2–3+ biomarkers had a 35% progression rate at 18 months compared to 6% for patients with 0–1+ biomarkers.

### 3.4. mALBI Grade as a Modifier for Progression Risk in Negative-1+ Biomarker Patients

**Figure 3:**
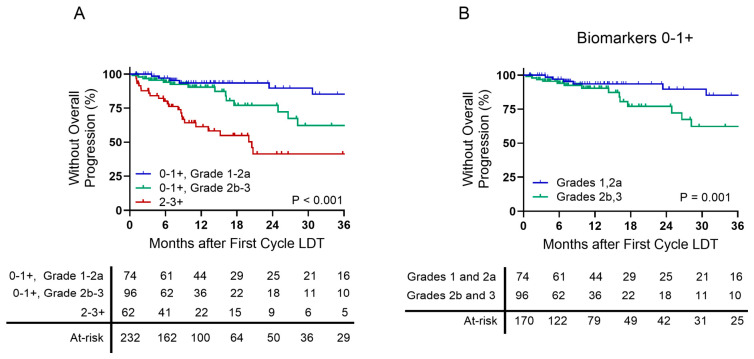
Overall time-to-progression based on mALBI grade and biomarker profile. (**A**) Overall TTP in patients with biomarker profiles of 0 or 1+ based on mALBI grade, and those with biomarker profiles of 2+ and 3+. (**B**) Overall TTP in patients, either triple negative or positive for a single biomarker at HCC diagnosis, based on mALBI grade.

Although mALBI was not associated with tumor burden within BCLC-A-stage disease, the delayed stratification of TTP risk could result in mALBI-based TTP stratification within discreet biomarker profiles. In complex biomarker profiles (2–3+), mALBI grade was not able to further stratify TTP, as these profiles continued to show unsatisfactory TTP (median 20 months) with 1– and 2–year progression rates of 39% and 59%, regardless of mALBI grade (Figure 3A, failure plots are shown in Supplemental Figure S3). mALBI grade was able to stratify progression risk in patients with 0–1+ biomarkers, wherein patients with mALBI grades of 2b–3 experienced higher progression risks than those with lower mALBI grades (1–2a) (Figure 3B). In low-complex biomarker profiles (0–1+), the 2-year progression risk of patients with grades 2b–3 was 23% compared to 10% for grades 1–2a, providing an additional method to identify post-treatment progression risk.

## 4. Discussion

Hepatic reserve and tumor burden have well-defined roles in HCC staging and overall prognosis [22]. Early-stage, BCLC-A staging provides the greatest access to surgical, liver-directed, and systemic therapy and therefore optimal OS projections exceeding 5 years [22]. However, several studies have highlighted that BCLC-A patients are at high risk of tumor progression following standard treatment approaches [6,23], likely due to aggressive biological responses in a manner that cannot be readily identified at initial diagnosis. While this aggressive response often manifests at primary treatment imaging follow-up (<3 months after treatment), aggressive disease may also initially present as an objective radiographic response but is then accompanied by a rapid onset of locally recurrent disease or extrahepatic spread less than 1 year after initial treatment. In the absence of routine, biopsy-based HCC characterization in early-stage disease, the ongoing breakthroughs in both blood-based and cell-free DNA biomarkers will ideally help identify these aggressive biological signatures, evolve staging algorithms, and expand treatment algorithms to improve overall outcomes.

In the current landscape of BCLC-A disease, the major prognostic indicators of aggressive biology include: stage-intrinsic measures of tumor burden (solitary/multifocal disease, cumulative tumor size) [22], preserved liver function reflected by albumin level or albumin-containing risk index score [24,25,26], and complex HCC biomarker expression profiles [16,21]. Unresectable, small, solitary HCC burden can be curatively treated with heat- or radiation-based LDT and potentially sequenced to curative surgery to address the risk of future cirrhosis-driven de novo disease [27,28]. Accordingly, more significant or multifocal disease presents additional challenges to complete ablative treatments that could increase progression risk before a complete radiographic response or surgical intervention is achieved. Both albumin and ALBI/mALBI have shown promise for tumor-indirect, potential immune-mediated OS prognosis and progression risk (Ref. [24], see review [8]), although the mechanisms of tumor-specific prognosis remain unclear. Finally, several groups have shown that the AFP, AFP-L3, and DCP biomarker system has tumor-direct prognostic implications beyond the surveillance/diagnosis realm [16,18,21,29].

Blood-count-based inflammatory cell ratios, NLR and PLR, are well described in the HCC literature for OS prognosis [30,31,32,33,34,35] and have an unclear role in direct tumor prognosis [10,32,35]. While limited evidence in some cancers suggests that the NLR may correlate with the intensity of the tumor-infiltrating lymphocyte [36], both NLR and PLR can exhibit high point-to-point variance in the setting of deteriorating liver function [37,38], viral/bacterial infections [39], and emergent hematopoiesis associated with terminal disease [35]. In early-stage disease, where performance status, preserved liver function, and tumor burden are more stable, PLRs were not associated with post-treatment disease progression risk. While two NLR cutoffs were prognostic for TTP, both fell within the normal range of healthy adults [40]. This suggests that NLR/PLR-based prognosis may be independent of tumor biology, and the critical role neutrophilia or lymphopenia may play in identifying immune exhaustion and immune-checkpoint inhibitor (ICI) non-responders.

HCC biomarker profiling at BCLC A-B diagnosis continues to show an ability to both stratify patients at risk of rapid disease progression as well as biomarker panel negative patients with favorable tumor biology [16,21]. Further, the current results additionally support the role of biomarker profiling maintained through BCLC-A sub-staging, capable of stratifying high-risk populations for otherwise optimal small, solitary diseases. Maintained stratification in BCLC-A sub-staging, and potentially the more diverse BCLC-B sub-stages, may have important implications in disease upstaging, including access to more aggressive combination therapies such as LDT + ICI [41]. Other recent studies in this patient population have shown AFP-L3 and DCP to be associated with undetectable microvascular invasion in solitary disease [42] as well as aggressive features not detectable in pre-transplant imaging but which are present in post-transplant pathology [29].

While the systemic inflammatory NLR and PLR were not associated with tumor progression risk, the mALBI, assessed at treatment baseline, displayed the ability to stratify longer-term progression risk 1–2 years after treatment, independent of the initial biomarker profile. One advantage of the mALBI over ALBI, particularly in the context of early-stage disease, is its ability to stratify a large population of patients in the ALBI-2 category into mALBI-2a and -2b. An earlier study by our group identified a tumor-burden-independent prognostic role of albumin in early-stage disease that was less evident using the ALBI system due to the large, diverse population of patients in ALBI-2 [24]. When mALBI stratification was applied within the ideal biomarker profiles (0–1+), an ideal patient population with a 3-year progression risk of 51% could be observed. The elevated progression risk in mALBI 2b-3 biomarker (0–1+) patients may be attributable to persistent partially responsive/maladaptive tumor burden or a new burden with more aggressive biology, particularly given the role of albumin in HCC development risk.

However, mALBI was unable to further stratify progression risk in patients with an aggressive biomarker profile (2–3+), suggesting that complex biomarker expression profiles may provide the best HCC-specific, clinically translatable strategy to identify aggressive tumor biology unresponsive to LDT. Biomarker profiling may also have similar implications in BCLC-B disease, where both disease and management strategies are more diverse, and BCLC-C disease, where strategies to predict treatment responder populations in the increasingly diverse ICI space have remained challenging. In the more challenging BCLC B-C space, the mALBI, and potentially NLR/PLR, may have important implications for identifying insufficient hepatic reserve or systemic manifestations of terminal disease, wherein immune-based treatments may be prohibitive. This also highlights the longitudinal implications of both biomarker profiling and mALBI, where declining mALBI grade or increasing biomarker profile [43] could identify post-treatment progression risk prior to routine follow-up imaging.

This single-center study prospectively analyzed patient outcomes based on biomarker profiling, HCC staging, and mALBI score at diagnosis and prior to first-cycle LDT. The center treatment algorithm evolved over the course of enrollment, including an early-adopter period involving ^90^Y transarterial radioembolization. While we did not observe a confounding role of treatment modality, provider-modality experience may shift overall progression rates in BCLC-A between different treatment centers. Emerging data suggests that longitudinal biomarker profiling may provide a superior assessment of short-term progression risk relative to baseline (treatment-naïve) profiles and warrants further investigation. While the GALAD has received a breakthrough device designation by the FDA, validation for widespread implementation is ongoing and may create a barrier to widespread clinical accessibility.

## 5. Conclusions

In conclusion, non-invasive biomarker profiling using AFP, AFP-L3, and DCP identifies clinical manifestations of aggressive tumor biology in early-stage disease and is directly associated with short-term, tumor-dependent prognosis. Measures of hepatic reserve such as the mALBI can refine longer-term, tumor-linked prognosis in patients with favorable HCC biomarker profiles in a manner that may be dependent upon albumin level. Peripheral immune cell ratios utilized as measures of systemic inflammation may not be reflective of tumor biology in otherwise early-stage disease but may remain effective for OS prognosis.

## Figures and Tables

**Table 1:** Study cohort demographics and characteristics at HCC diagnosis.

Demographic	Cohort
Patients, n (%)	232
Follow-up to Primary Endpoint, months and median (IQR)	9.8 (5–20)
Overall Study Follow-up, months and median (IQR)	27 (12–56)
Age at HCC Diagnosis, years and median (IQR)	64 (60–68)
Sex, self-reported, male n (%)	162 (70)
Race, self-reported, n (%)	
Caucasian/White	180 (78)
African American/Black	43 (18)
Other	9 (4)
Cirrhotic Etiology, n (%)	
Viral	145 (63)
SLD	76 (32)
Other	11 (5)
Scores and Staging	
ECOG Performance Status, n (%)	
Score 0	161 (71)
Score 1	65 (29)
Child–Pugh, n (%)	
A	141 (61)
B	75 (32)
C	16 (7)
Clinical Labs at Diagnosis	
Sodium, mM, median (IQR)	139 (137–141)
Creatinine, mg/dL, median (IQR)	0.9 (0.8–1.1)
Bilirubin, mg/dL, median (IQR)	1.0 (0.6–1.7)
Albumin, g/dL, median (IQR)	3.4 (2.9–3.8)
ALBI Grade, n (%)	
1	42 (18)
2	153 (66)
3	37 (16)
mALBI Grade, n (%)	
1	42 (18)
2a	52 (22)
2b	101 (44)
3	37 (16)
INR, ratio and median (IQR)	1.1 (1.0–1.2)
MELD 3.0, score (IQR)	10 (8–13)
White Blood Cell Count, 10^3^/μL, median (IQR)	5.2 (3.8–6.8)
Absolute Neutrophil Count, 10^3^/μL, median (IQR)	2.9 (2.1–4.2)
Absolute Lymphocyte Count, 10^3^/μL, median (IQR)	1.4 (0.9–2.0)
Platelets, 10^3^/μL, median (IQR)	108 (72–175)
NLR, median (IQR)	2.1 (1.5–3.1)
Unable to access, n (%)	2 (<1)
PLR, median (IQR)	82 (58–120)
Unable to access, n (%)	1 (<1)
HCC Burden and Biomarkers	
BCLC 2022 Stage, n (%)	
A	232 (100)
Multifocal, n (%)	
Solitary	197 (85)
Index Lesion Diameter, cm, median (IQR)	2.8 (2.2–3.7)
Cumulative Lesion Size, cm, median (IQR)	3.3 (2.3–4.2)
≤3 cm	124 (53)
>3 cm	108 (47)
HCC Biomarkers	
AFP, ng/mL, median (IQR)	7.7 (4.5–51)
AFP-L3, %, median (IQR)	5.3 (0.5–20)
DCP, ng/mL, median (IQR)	2.6 (1.5–7.6)
HCC Biomarker Profile	
0	107 (46)
1+	63 (27)
2+	42 (18)
3+	20 (9)
Liver-Directed Therapy	
LDT Modality, n (%)	
DEB-TACE	44 (19)
MWA	51 (22)
90Y	137 (59)

Abbreviations: Interquartile range (IQR), Hepatocellular carcinoma (HCC), Steatotic liver disease (SLD), Eastern Cooperative Oncology Group (ECOG), International normalized ratio (INR), Liver-directed therapy (LDT), Albumin–bilirubin (ALBI), Modified albumin–bilirubin (mALBI), Neutrophil-to-lymphocyte ratio (NLR), Platelet-to-lymphocyte ratio (PLR), Doxorubicin-eluting bead transarterial chemoembolization (DEB-TACE), Microwave ablation (MWA), Yttrium-90 (90Y), Model end-stage liver disease (MELD), Barcelona Clinic Liver Cancer (BCLC), Alpha-fetoprotein (AFP), AFP-Lens culinaris agglutinin (AFP-L3), Des-gamma-carboxy prothrombin (DCP).

**Table 2:** Cox proportional hazards of variables associated with time to progression to advanced stages.

Demographic	Univariate HR (95% CI)	*p*-Value	Multivariate HR (95% CI)	*p*-Value
Age at HCC Diagnosis		0.905		
Sex, self-reported		0.636		
Race, self-reported		0.210		
Cirrhotic Etiology		0.541		
Scores and Staging				
ECOG Performance Status		0.454		
Child–Pugh		0.095		
Clinical Labs at Baseline				
Sodium, mM		0.849		
Creatinine, mg/dL		0.540		
Bilirubin, mg/dL		0.166		
Albumin, g/dL	0.44 (0.25–0.78)	0.005		
ALBI Grade		0.080		
mALBI Grade				
1 and 2a vs. 2b and 3	0.38 (0.20–0.73)	0.002	0.45 (0.23–0.89)	0.021
INR, ratio		0.366		
MELD 3.0		0.185		
White Blood Cell Count, 10^3^/μL		0.706		
Absolute Neutrophil Count, 10^3^/μL		0.334		
Absolute Lymphocyte Count, 10^3^/μL		0.105		
Platelets, 10^3^/μL		0.885		
NLR, median cutoff		0.140		
≥2.1 vs. <2.1				
NLR, literature cutoffs				
>2.81 vs. <2.81	1.9 (1.1–3.5)	0.033		0.258
>3.0 vs. <3.0	1.9 (1.1–3.6)	0.033		0.468
PLR, median cutoff		0.432		
≥ 82 vs. < 82				
HCC Burden and Biomarkers				
Multifocal, n (%)		0.706		
Index Lesion Diameter, cm	1.3 (1.1–1.5)	<0.001		
Cumulative Lesion Size, cm	1.3 (1.1–1.5)	<0.001		
>3 cm vs. ≤3 cm	3.3 (1.7–7.0)	<0.001	2.8 (1.4–5.8)	0.005
HCC Biomarker Profile		<0.001		<0.001
0 and 1+ vs 2+	0.33 (0.17–0.66)	0.002	0.38 (0.19–0.75)	0.005
0 and 1+ vs 3+	0.12 (0.06–0.27)	<0.001	0.21 (0.09–0.46)	<0.001
2+ vs. 3+	0.37 (0.16–0.85)	0.018		0.166
Liver-Directed Therapy				
LDT Modality, n (%)		0.145		

Abbreviations: Hepatocellular carcinoma (HCC), Eastern Cooperative Oncology Group (ECOG), International normalized ratio (INR), Liver-directed therapy (LDT), Albumin–bilirubin (ALBI), Modified albumin–bilirubin (mALBI), Neutrophil-to-lymphocyte ratio (NLR), Platelet-to-lymphocyte ratio (PLR), Model end-stage liver disease (MELD).

## Data Availability

All data included in this study are available upon reasonable request to the corresponding author.

## Supplementary Materials

The following supporting information can be downloaded at https://www.mdpi.com/article/10.3390/cancers18132073/s1. Table S1: Cox proportional hazards of mALBI, cumulative lesion size, biomarker combinations associated with time to stage progression; Table S2: univariate analysis of time to stage progression based on NLR cutoffs for HCC across BCLC stages; Table S3: univariate analysis of time to stage progression based on PLR cutoffs for HCC across BCLC stages; Table S4: relationship between cumulative lesion size, mALBI, and biomarker profile. Table S5. Relationship between Cumulative Lesion Size, mALBI, and Biomarker Profile. Figure S1. Overall Time-to-Progression based on HCC Biomarker Expression Profile and mALBI Grade. Overall TTP failure plots following first cycle liver-directed therapy based on (A) HCC biomarkers profiles, (B) mALBI grade, and (C) cumulative lesion size grouped at the time of HCC diagnosis. Figure S2. Overall Time-to-Progression by Cumulative Lesion Size based on the Number of Positive HCC Biomarkers. (A) Overall TTP failure plots in patients with initial cumulative lesion sizes of either (A) > 3 cm or (B) ≤ 3 cm based on HCC biomarker profiles at the time of HCC diagnosis. Figure S3. Overall Time-to-Progression based on mALBI Grade and Biomarker Profile. (A) Overall TTP failure plots in patients with biomarker profiles of 0 or 1+ based on mALBI grade, and those with biomarker profiles of 2+ and 3+. (B) Overall TTP failure plots in patients either triple negative or positive for a single biomarker at HCC diagnosis based on mALBI grade.

## Abbreviations

The following abbreviations are used in this manuscript:

HCC	Hepatocellular carcinoma
LDT	Liver-directed therapy
BCLC	Barcelona Clinic Liver Cancer
DEB-TACE	Doxorubicin-eluting bead transarterial chemoembolization
TARE	Transarterial radioembolization
90Y	Yttrium-90
ALBI	Albumin–bilirubin
mALBI	Modified albumin–bilirubin
NLR	Neutrophil–lymphocyte ratio
PLR	Platelet–lymphocyte ratio
AFP	Alpha-fetoprotein
AFP-L3	AFP-Lens culinaris agglutinin fraction
DCP	Des-gamma-carboxy prothrombin
TTP	Time to progression
OS	Overall survival
