# Supplementary material for: Baseline Tumor-Specific Prognosis in Early-Stage Hepatocellular Carcinoma: Time-Dependent Role of Biomarker Profile and Modified ALBI Grade
Source: Cancers (Basel). 2026 Jun 26;18(13):2073. doi: 10.3390/cancers18132073 (PMC13359626; doi:10.3390/cancers18132073)
Supplement: Supplementary file 1 [file cancers-18-02073-s001.zip › cancers-4374959-supplementary.pdf]

SUPPLEMENTAL FIGURE

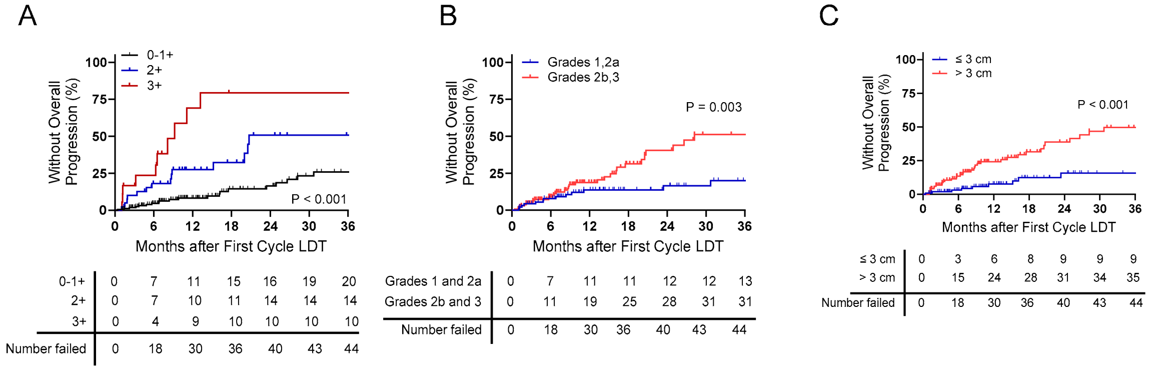

Supplemental Figure S1. Overall Time-to-Progression based on HCC Biomarker Expression Profile and mALBI Grade. Overall TTP failure plots following first cycle liver-directed therapy based on (A) HCC biomarkers profiles, (B) mALBI grade, and (C) cumulative lesion size grouped at the time of HCC diagnosis.

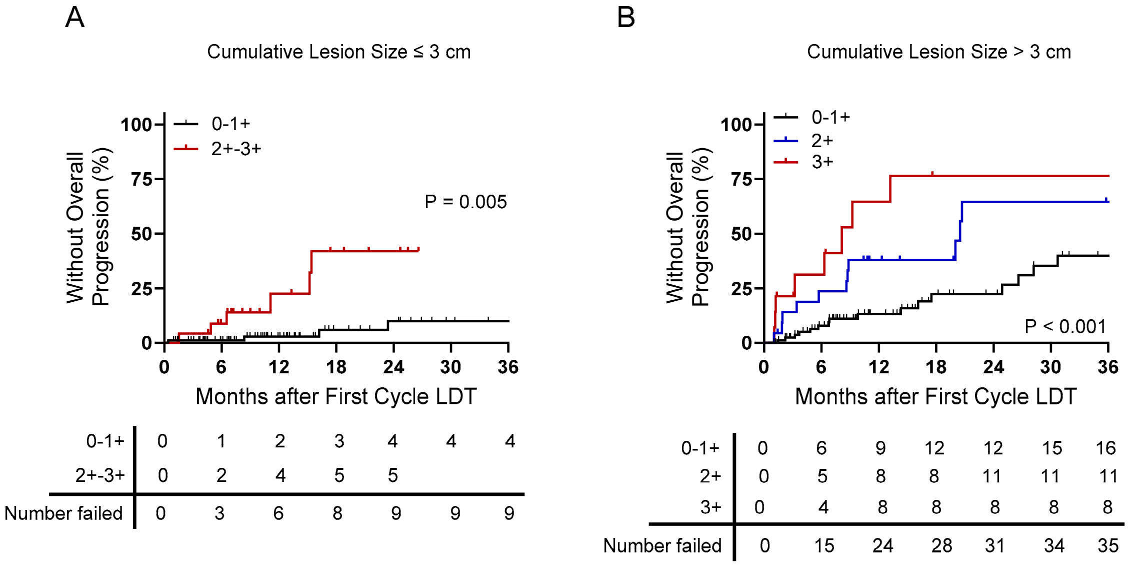

Supplemental Figure S2. Overall Time-to-Progression by Cumulative Lesion Size based on the Number of Positive HCC Biomarkers. (A) Overall TTP failure plots in patients with initial cumulative lesion sizes of either (A) > 3cm or (B) ≤ 3 cm based on HCC biomarker profiles at the time of HCC diagnosis.

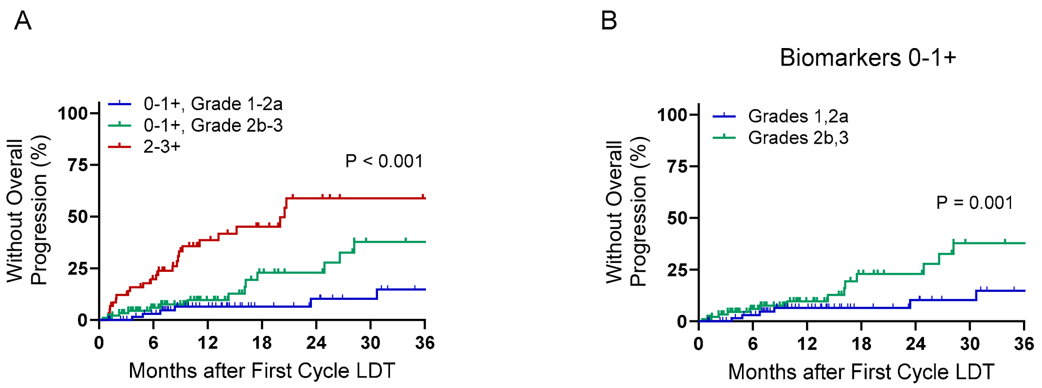

0-1+, Grade 1-2a	0	2	4	4	5	5	6
0-1+, Grade 2b-3	0	5	7	11	11	14	14
2-3+	0	11	19	21	24	24	24
Number failed	0	18	30	36	40	43	44

Grades 1 and 2a	0	2	4	4	5	5	6
Grades 2b and 3	0	5	7	11	11	14	14
Number failed	0	7	11	15	16	19	20

Supplemental Figure S3. Overall Time-to-Progression based on mALBI Grade and Biomarker Profile. (A) Overall TTP failure plots in patients with biomarker profiles of 0 or 1+ based on mALBI grade, and those with biomarker profiles of 2+ and 3+. (B) Overall TTP failure plots in patients either triple negative or positive for a single biomarker at HCC diagnosis based on mALBI grade.

SUPPLEMENTAL TABLES

**Supplemental Table S1. Time to Advanced Stage Progression Outcomes**	
**Status at Data Analysis, n (% of total)**	**Cohort (n = 232)**
Active	42 (18)
Censored	73 (31)
Censored due to transplantation	71 (31)
Progression to Advanced Stage	46 (20)

**Supplemental Table S2. Cox Proportional Hazards of mALBI, Cumulative Lesion Size, Biomarker Combinations Associated with Time to Stage Progression**		
**Variables**	**Univariate HR (95% CI)**	**P-Value**
**mALBI Grade**		**0.022**
1 vs 2a	0.75 (0.25 – 2.3)	0.622
1 vs 2b	0.33 (0.13 – 0.87)	**0.024**
1 vs 3	0.28 (0.08 – 0.95)	**0.041**
2a vs 2b	0.44 (0.20 – 0.98)	**0.044**
2a vs 3	0.37 (0.13 – 1.1)	0.075
2b vs 3	0.85 (0.34 – 2.1)	0.715
**Cumulative Lesion Size, Group 1**		**0.003**
< 3 cm vs 3 – 5 cm	0.37 (0.18 – 0.77)	**0.008**
< 3 cm vs > 5 cm	0.25 (0.10 – 0.63)	**0.003**
3 – 5 cm vs > 5 cm	0.68 (0.32 – 1.5)	0.323
**Cumulative Lesion Size, Group 2**		
> 3 cm vs ≤ 3 cm	3.5 (1.7 – 7.0)	**<0.001**
**HCC Biomarker Profile**		**<0.001**
0 vs 1+	0.49 (0.21 – 1.1)	0.100
0 vs 2+	0.24 (0.10 – 0.55)	**<0.001**
0 vs 3+	0.09 (0.04 – 0.22)	**<0.001**
1+ vs 2+	0.48 (0.22 – 1.0)	0.062
1+ vs 3+	0.18 (0.08 – 0.42)	**<0.001**
2+ vs 3+	0.37 (0.16 – 0.85)	**0.018**
**Abbreviations: Hepatocellular carcinoma (HCC), Modified Albumin-Bilirubin (mALBI).**		

**Supplemental Table S3. Univariate Analysis of Time to Stage Progression based on NLR Cutoffs for HCC Across BCLC Stages**								
**Study**	**No. of patients**	**Cirrhosis**	**BCLC Stage**	**BCLC Breakdown**	**Etiology**	**Therapy**	**NLR Cutoff**	**P-Value**
**This study[a]**	237	100%	A	100% A	63% Viral	DEB-TACE/Ablation/^{90}Y	≥ 2.1	0.140
**Lu et al. 2016[1]**	963	83%	0/A/B/C	63% 0/A	85% HBV	Resection	> 2.81	**0.037**
**Wong et al. 2019[2]**	789	N/A	0/A/B/C/D	41% A	43% HCV	Resection	≥ 3.0	**0.041**
**Schobert et al. 2020[3]**	46	100%	A/B/C	30% B	39% HCV	DEB-TACE	≥ 3.22	0.075
**Minici et al. 2024[4]**	214	99.5%	B	100% B	43% HCV	DEB-TACE	≥ 4.72	0.862
**Sukato et al. 2015[5]**	176	58.5%	B/C	56% B	39% Viral	^{90}Y	≥ 5.0	0.594

Abbreviations: Hepatocellular carcinoma (HCC), Hepatitis C virus (HCV), Hepatitis B virus (HBV), Doxorubicin-eluting beads transarterial chemoembolization (DEB-TACE), Yttrium-90 (^{90}Y), Barcelona Clinic Liver Cancer (BCLC), Neutrophil-to-lymphocyte ratio (NLR).
[a] Median NLR of cohort was used.

**Supplemental Table S4. Univariate Analysis of Time to Stage Progression based on PLR Cutoffs for HCC Across BCLC Stages**								
**Study**	**No. of patients**	**Cirrhosis**	**BCLC Stage**	**BCLC Breakdown**	**Etiology**	**Therapy**	**PLR Cutoff**	**P-Value**
**Kyung Bae et al. 2022[6]**	4076	N/A	0/A	100% 0/A	88% Viral	Transplant/Resection/Ablation/TACE/TARE	> 65	0.893
**This study[a]**	237	100%	A	100% A	63% Viral	DEB-TACE/Ablation/^{90}Y	≥ 82	0.432
**Young et al. 2021[7]**	296	100%	N/A	N/A	49% HCV	TACE	> 88.3	0.173
**Schobert et al. 2020[3]**	46	100%	A/B/C	30% B	39% HCV	DEB-TACE	≥ 113.1	0.083
**Minici et al. 2024[4]**	214	99.5%	B	100% B	43% HCV	DEB-TACE	≥ 119.7	0.123

Abbreviations: Hepatocellular carcinoma (HCC), Hepatitis C virus (HCV), Hepatitis B virus (HBV), Doxorubicin-eluting beads transarterial chemoembolization (DEB-TACE), Yttrium-90 (^{90}Y), Barcelona Clinic Liver Cancer (BCLC), Platelet-to-lymphocyte ratio (PLR), Not available (N/A).
a Median PLR of cohort was used.

**Supplemental Table S5. Relationship between Cumulative Lesion Size, mALBI, and Biomarker Profile**			
	**Cumulative Lesion Size**		
**Variables**	**≤ 3 cm**	**> 3 cm**	**P-Value**
**mALBI Grade, n (%)**			0.839
1 and 2a	43 (40)	51 (41)	
2b and 3	65 (60)	73 (59)	
**Biomarker Profile, n (%)**			**0.035**
0 – 1+	84 (78)	86 (69)	
2+	20 (19)	22 (18)	
3+	4 (3)	16 (13)	
	**mALBI Grade**		
**Variables**	**1 and 2a**	**2b and 3**	
**Biomarker Profile, n (%)**			0.102
0 – 1+	74 (79)	96 (70)	
2+	16 (17)	26 (19)	
3+	4 (4)	16 (11)	
**Abbreviations: Hazard ratio (HR), Confidence interval (CI), Modified albumin-bilirubin (mALBI), Hepatocellular carcinoma (HCC).**			

REFERENCES

1. Lu, S.D.; Wang, Y.Y.; Peng, N.F.; Peng, Y.C.; Zhong, J.H.; Qin, H.G.; Xiang, B.D.; You, X.M.; Ma, L.; Li, L.Q. Preoperative Ratio of Neutrophils to Lymphocytes Predicts Postresection Survival in Selected Patients With Early or Intermediate Stage Hepatocellular Carcinoma. *Medicine (Baltimore)* **2016**, *95*, e2722, doi:10.1097/MD.0000000000002722.
2. Wong, L.; Bozhilov, K.; Hernandez, B.; Kwee, S.; Chan, O.; Ellis, L.; LeMarchand, L. Underlying liver disease and advanced stage liver cancer are associated with elevated neutrophil-lymphocyte ratio. *Clin Mol Hepatol* **2019**, *25*, 305-316, doi:10.3350/cmh.2019.0004.
3. Schobert, I.T.; Savic, L.J.; Chapiro, J.; Bousabarah, K.; Chen, E.; Laage-Gaupp, F.; Tefera, J.; Nezami, N.; Lin, M.; Pollak, J.; et al. Neutrophil-to-lymphocyte and platelet-to-lymphocyte ratios as predictors of tumor response in hepatocellular carcinoma after DEB-TACE. *Eur Radiol* **2020**, *30*, 5663-5673, doi:10.1007/s00330-020-06931-5.
4. Minici, R.; Venturini, M.; Guzzardi, G.; Fontana, F.; Coppola, A.; Piacentino, F.; Torre, F.; Spinetta, M.; Maglio, P.; Guerriero, P.; et al. A Multicenter International Retrospective Investigation Assessing the Prognostic Role of Inflammation-Based Scores (Neutrophil-to-Lymphocyte, Lymphocyte-to-Monocyte, and Platelet-to-Lymphocyte Ratios) in Patients with Intermediate-Stage Hepatocellular Carcinoma (HCC) Undergoing Chemoembolizations of the Liver. *Cancers (Basel)* **2024**, *16*, doi:10.3390/cancers16091618.
5. Sukato, D.C.; Tohme, S.; Chalhoub, D.; Han, K.; Zajko, A.; Amesur, N.; Orons, P.; Marsh, J.W.; Geller, D.A.; Tsung, A. The Prognostic Role of Neutrophil-to-Lymphocyte Ratio in Patients with Unresectable Hepatocellular Carcinoma Treated with Radioembolization. *J Vasc Interv Radiol* **2015**, *26*, 816-824 e811, doi:10.1016/j.jvir.2015.01.038.
6. Bae, B.K.; Park, H.C.; Yoo, G.S.; Choi, M.S.; Oh, J.H.; Yu, J.I. The Significance of Systemic Inflammation Markers in Intrahepatic Recurrence of Early-Stage Hepatocellular Carcinoma after Curative Treatment. *Cancers (Basel)* **2022**, *14*, doi:10.3390/cancers14092081.
7. Young, S.; Cam, I.; Gencturk, M.; Rubin, N.; D'Souza, D.; Flanagan, S.; Golzarian, J.; Sanghvi, T. Inflammatory Scores: Comparison and Utility in HCC Patients Undergoing Transarterial Chemoembolization in a North American Cohort. *J Hepatocell Carcinoma* **2021**, *8*, 1513-1524, doi:10.2147/JHC.S335183.
